# Inhibition of Advanced Glycation End Products in Yogurt by Lotus Seedpod Oligomeric Procyanidin

**DOI:** 10.3389/fnut.2021.781998

**Published:** 2021-11-04

**Authors:** Nianjie Feng, Yang Shen, Chuanqin Hu, Jiangying Tan, Zhao Huang, Chao Wang, Zhiqiang Guo, Qian Wu, Juan Xiao

**Affiliations:** ^1^Key Laboratory of Fermentation Engineering (Ministry of Education), Hubei Key Laboratory of Industrial Microbiology, National “111” Center for Cellular Regulation and Molecular Pharmaceutics, Hubei Research Center of Food Fermentation Engineering and Technology, Hubei University of Technology, Wuhan, China; ^2^Key Laboratory of Cleaner Production and Integrated Resource Utilization of China National Light Industry, Beijing Technology and Business University, Beijing, China; ^3^State Key Laboratory of Marine Resource Utilization in South China Sea, Key Laboratory of Food Nutrition and Functional Food of Hainan Province, Ministry of Education, Engineering Research Center of Utilization of Tropical Polysaccharide Resources, School of Food Science and Engineering, Hainan University, Haikou, China

**Keywords:** yogurt, lotus seedpod oligomeric procyanidin, advanced glycation end products, physicochemical properties, flavor

## Abstract

The basic ingredients of yogurt include lactose and protein. Yogurt undergoes the Maillard reaction easily, producing many advanced glycation end products (AGEs) that cause some chronic diseases. Lotus seedpod oligomeric procyanidin (LSOPC) have demonstrated a strong inhibitory effect on AGE formation in simulated models; however, the inhibition of procyanidin on AGE formation and the subsequent effects on yogurt quality remains unknown. Our study demonstrated that LSOPC had a good inhibitory effect on the formation of fluorescent AGEs and Nε-carboxymethyl lysine (*P* < 0.05). The inhibitory capacity on AGEs and antioxidant activity of yogurt were positively correlated with the concentration of LSOPC. The effect of LSOPC on the physicochemical properties of yogurt was also evaluated. Bound water content, viscosity, and flavor of yogurt were significantly increased after LSOPC addition (*P* < 0.05). Therefore, LSOPC may lead to significant benefits for controlling AGE formation and improving the quality of yogurt.

## Introduction

Yogurt is a fermented dairy food produced by the growth of lactic acid bacteria (LAB). Due to the antioxidant peptides generated during fermentation, yogurt has higher antioxidant properties than ordinary milk (1). Meanwhile, proteins are degraded into small-molecule compounds (2) that are more easily digested and absorbed. In addition, yogurt contains large amounts of amino acids, such as lysine, tryptophan, and methionine, and reducing sugars, such as glucose, galactose, and lactose. Then, free amino groups and reducing sugars interact and undergo non-enzymatic protein glycation, forming Schiff base and Amadori products. Subsequently, various dicarbonyl compounds are produced through the oxidation and dehydration of the Amadori products to react with amino groups to form advanced glycation end products (AGEs) (3). The AGEs content of yogurt (vanilla flavor) is 3.00 kU/100 ml.

AGEs are produced by complex Maillard reactions. AGEs, including Nε-carboxymethyl lysine (CML), methylglyoxal (MGO), and pentosidine, are a group of potentially harmful compounds. Humans are exposed to AGEs in two ways, via endogenous AGEs generated from abnormal glucose metabolism or lipid peroxidation or via exogenous AGEs from various foods that they consume daily. Exogenous AGEs can enter the bloodstream and grow in the body (4). The AGEs accumulated in the body can increase oxidative stress, activate nuclear factor-κB, further inducing various cytokines and growth factors. In any case, AGEs have been proposed to be a risk factor in the pathogenesis of diet-related diseases, such as diabetes, insulin resistance, cardiovascular diseases, kidney injury, and age-related neurodegenerative diseases (5).

Thus, the amount of AGEs in yogurt needs to be controlled. Our previous studies have shown that procyanidin can inhibit AGE formation in simulated food processing (6, 7). Procyanidin, a type of polyphenols, are widely distributed in the plant kingdom (8). Due to their special chemical structures, procyanidin display strong antioxidant properties and free radical scavenging abilities, similar to condensed tannins or oligomeric flavonoids (9–12). Many studies have shown that procyanidin can provide several health benefits, such as preventing cancer and cardiovascular diseases (11–13), reducing acrylamide content in food (14), and resisting neonatal hypoxic-ischemic brain damage (15, 16). Yogurt is a complex system considering its chemical constitution and physical environment. The inhibition of procyanidin on AGE formation and the subsequent effects on yogurt quality remain unclear.

In this study, a B-type procyanidin, named lotus seedpod oligomeric procyanidin (LSOPC), was extracted from lotus seed waste. LSOPC, as a yogurt additive, was found to inhibit AGE formation. The effect of LSOPC on the flavor and physicochemical properties of yogurt was also investigated.

## Materials and Methods

### Materials

Milk (Weidendorf, Germany), caster sugar (Chuan Xiu, Beijing, China), and starter cultures *Lactobacillus plantarum* 21784 (China Industrial Microbial Culture Preservation Management Center) were purchased. Ultrapure water was obtained with a Milli-Q water purification system (Millipore, Microsep, Bellville, USA). All other chemicals (Sinopharm Chemical Reagent Factory, Shanghai, China) were analytical grade. Oligomeric procyanidin were prepared from lotus seedpods (a local supermarket, Wu Zhi 2 hao, China), according to Wu et al. (4).

### Yogurt Preparation

Yogurts were prepared as described by Cenobio-Galindo et al. (17). In summary, 100 g milk and 5 g caster sugar were mixed with various concentrations of LSOPC and pasteurized at 85°C for 30 min. 0.2 g of starter culture was inoculated into the mixture, and maintained at 37°C for 12 h.

### Counting *Lactobacillus plantarum*

The spread plate method was used to count *L. plantarum* (18). In summary, the sample was diluted with saline to the desired dilution factor and incubated on MRS medium at 37°C for 48 h. Then, the number of colonies are counted.

### Titratable Acidity Determination

A 10-g sample was mixed with 20 mL of water and titrated with 0.1 M NaOH using phenolphthalein as an indicator (19). The control was under the same conditions without the sample. The acidity was calculated as:


(1) $$Acidity\;({}^{\circ}T)=\frac{C\times (V_{sample}-V_{control})\times 100}{m\times 0.1}$$

V_*sample*_ (mL) and V_*control*_ (mL) were the titration volume of sodium hydroxide for the sample and the control, respectively. C (in M) was the concentration of sodium hydroxide, m (in g) was the dry weight of the sample.

### Determination of the Fluorescent AGEs

All the samples were incubated at 50°C for 5 days and diluted 15 times to assess the formation of fluorescent AGEs using an F-4500 luminescence spectrometer (Shimadzu, Japan) at the excitation wavelength of 370 nm and emission wavelength of 440 nm (7). The control was a sample without LSOPC, and the blank was a sample without heating. The inhibition was calculated as:


(2) $$\begin{array}{l} Inhibition\;\left(\%\right)=\frac{A_{control}-A_{sample}}{A_{control}-A_{blank}}\times 100 \end{array}$$

A_*sample*_, A_*control*_, and A_*blank*_ were the absorbance of the sample, the control, and the blank, respectively.

### Nε-Carboxymethyl Lysine Determination

After incubation at 50°C for 5 days, 500 μL of a sample was added to 500 μL of 0.2 M sodium borohydride (pH 13, prepared with 0.1 M NaOH), and the mixture was maintained at 4°C for 10 h. The supernatant was collected by centrifugation and passed through a preactivated solid-phase extraction PCX column (20). The eluted compound was resuspended in 1 mL of 0.1% formic acid and filtered through a 0.22-μm organic membrane before HPLC-MS^2^ analysis. Three parallel experiments were performed.

Next, 15-μL samples were injected into an Eclipse Plus C_18_ column (2.1 × 50 mm, 5-μm, Agilent Technologies, Germany) at 30°C with 0.2% formic acid (solvent A) and acetonitrile (solvent B) serving as the mobile phases. The chromatographic conditions were optimized to a run time of 25 min and a flow rate of 0.2 mL/min to achieve good separation. The gradient program was as follows: 0–0.5 min, 90% A; 0.5–4.0 min, 90%−60% A; and 4.0–25.0 min, 60% A. The mass spectrometer with multiple reaction monitoring was operated in the positive ion mode. The nitrogen temperature was kept at 300°C and the capillary voltage at 4 kV. With MassHunter Data and MassHunter Qualitative (Agilent Technologies, Germany), the fragments at m/z 84 and 130 were used for the quantitative and qualitative analysis of CML (m/z 205), respectively.

### LSOPC Degradation Analysis

A UV spectrometer was used to measure the concentration of LSOPC at 546 nm (21). Standard compounds were used under the same conditions to prepare the standard curves. The degradation was calculated as:


(3) $$\begin{array}{l} Degradation\;\left(\%\right)=\frac{C_{a}-C_{b}}{C_{b}}\times 100 \end{array}$$

C_*a*_ and C_*b*_ were the concentration of LSOPC with and without treatment, respectively.

### Antioxidant Activity Analysis

A UV spectrometer was used to determine the radical scavenging activity using the 2,2-diphenyl-1-picrylhydrazyl (DPPH) method (22). In summary, 0.2 mL of a sample was added to 3.8 mL of 0.1 mM DPPH in ethanol. The mixture was incubated at room temperature for 30 min and measured at 517 nm. An ethanol solution without DPPH was used as the control group, and the blank group contained water instead of a sample. The DPPH radical scavenging activity was calculated as:


(4) $$\begin{array}{l} DPPH\;radical\;scavenging\;activity\;(\%)\;\\ =\frac{A_{blank}-(A_{sample}-A_{control})}{A_{blank}}\;\times \;100 \end{array}$$

A_*sample*_, A_*control*_, and A_*blank*_ were the absorbance of the sample, the control and the blank, respectively.

### Nuclear Magnetic Resonance

The transverse relaxation time (T_2_) of the sample was measured using the Carr-Purcell-Meiboom-Gill pulse sequence (23) and the following parameters: spectral width = 100 kHz, radio frequency delay = 0.16 ms, recycle delay = 3,000 ms, regulate analog gain 1 = 10, regulate digital gain 1 = 3, pre-amplified receiver gain = 1, scanning number = 8, echo number = 4,800, pulse gaps between π/2 and π = 0.16 ms.

### Rheological Property Determination

A DHR-3 rotational rheometer (TA Instruments Inc., United States) was applied to determine the dynamic rheological properties of the samples. The test conditions included the plate diameter of 40 mm, the gap of 0.5 mm, the temperature at 25°C, the scanning strain set to 1%, and the frequency at 0.1–10 Hz. Finally, the spectra of the storage modulus (G') and loss modulus (G”) were obtained.

The viscosity of yogurt was measured by the rotation method. All the samples were tested at 25 ± 0.5°C using a No. 3 rotor by a Brookfield DV-3T Rheology tester at the rotating speed of 60 rpm.

### Texture Determination

A TA.XT Plus texture analyzer with a P50 type probe (Stable Micro System Co., United States) was used to determine the texture of the samples (24) at the pretest, test, and post-test speeds of 1.0 mm/s and the trigger force of 5.0 g. The TPA software was used to analyze the final results.

### Gas Chromatography-Mass Spectrometry Analysis

A commercial solid-phase microextraction fiber with an 85-μm carbowax/polydimethylsiloxane coating (Supelco, Bellefonte, USA) was fitted into a manual holder and placed into the headspace above the sample to extract the flavor components at 50°C for 30 min (25). The fiber was then inserted into the needle and immediately injected into the GC-MS system. Desorption was performed at 250°C for 5 min.

The subsequent analyses were conducted using an Agilent 6890 GC system coupled to an Agilent 5975 inter quadrupole mass spectrometer. Sample components were separated on an Rtx-WAX capillary column (30 m × 0.25 mm, 0.25-mm film thickness; Agilent Technologies). The oven temperature program was set at 30°C for 3 min, to increase to 225°C at 15°C/min, and to hold for 5 min. The flow rate of the carrier gas (helium at ≥99.999% purity) was set at 1 mL/min. The mass selective detector was operated in the electron ionization mode at 70 eV over a scan range of m/z 30–500. The temperatures of the ion source and quadrupole were 240 and 150°C, respectively. Compound identification was based on mass spectra matching with the standard NIST 2001 MS library and on comparison to the retention indices sourced from the NIST Standard Reference Database and the authentic reference standards when available.

### Electronic-Nose Data Acquisition

A commercial PEN3 E-nose (Airsense Analytics GmbH, Schwerin, Germany) was used (26). A 1-mL sample was kept in a sealed bottle and heated at a selected temperature (70–150°C) for 1 h. Afterward, a 2 mL headspace was drawn off and injected into the e-nose at a flow rate of 300 mL/min. The response values were then recorded at 1-s intervals for 100 s until reaching a stable state. Finally, the probe was cleaned for 120 s, and the baseline was reset in 5 s. All the tests were performed at 25 ± 0.5°C.

### Organic Acid Analysis

Two g of sample was extracted by stirring with 25 mL of meta-phosphoric acid at 25°C at 150 rpm for 45 min and subsequently centrifuged at 10,000 rpm for 10 min (27). Before analysis by high-performance liquid chromatography (HPLC, Shimadzu, Kyoto, Japan), the sample was passed through a 0.45-μm nylon filter. The fractions were achieved on a reserved-phase eclipse plus C_18_ column (2.1 × 50 mm, 5-μm; Agilent Technologies, Germany) and eluted with 100 mM K_2_HPO_4_ in methanol (v/v, 97/3) using a flow rate of 1.0 mL/min. Detection was carried out in a PDA using 210 nm as a preferred wavelength.

### Statistical Analysis

All the data were presented as means ± standard deviation (means ± S.D.) and calculated using one-way ANOVA with SPSS 25.0 followed by the Tukey's multiple-range test. The graphs were drawn with OriginPro 8.0.

## Results and Discussion

### Analysis of LSOPC

**Figure 1:**
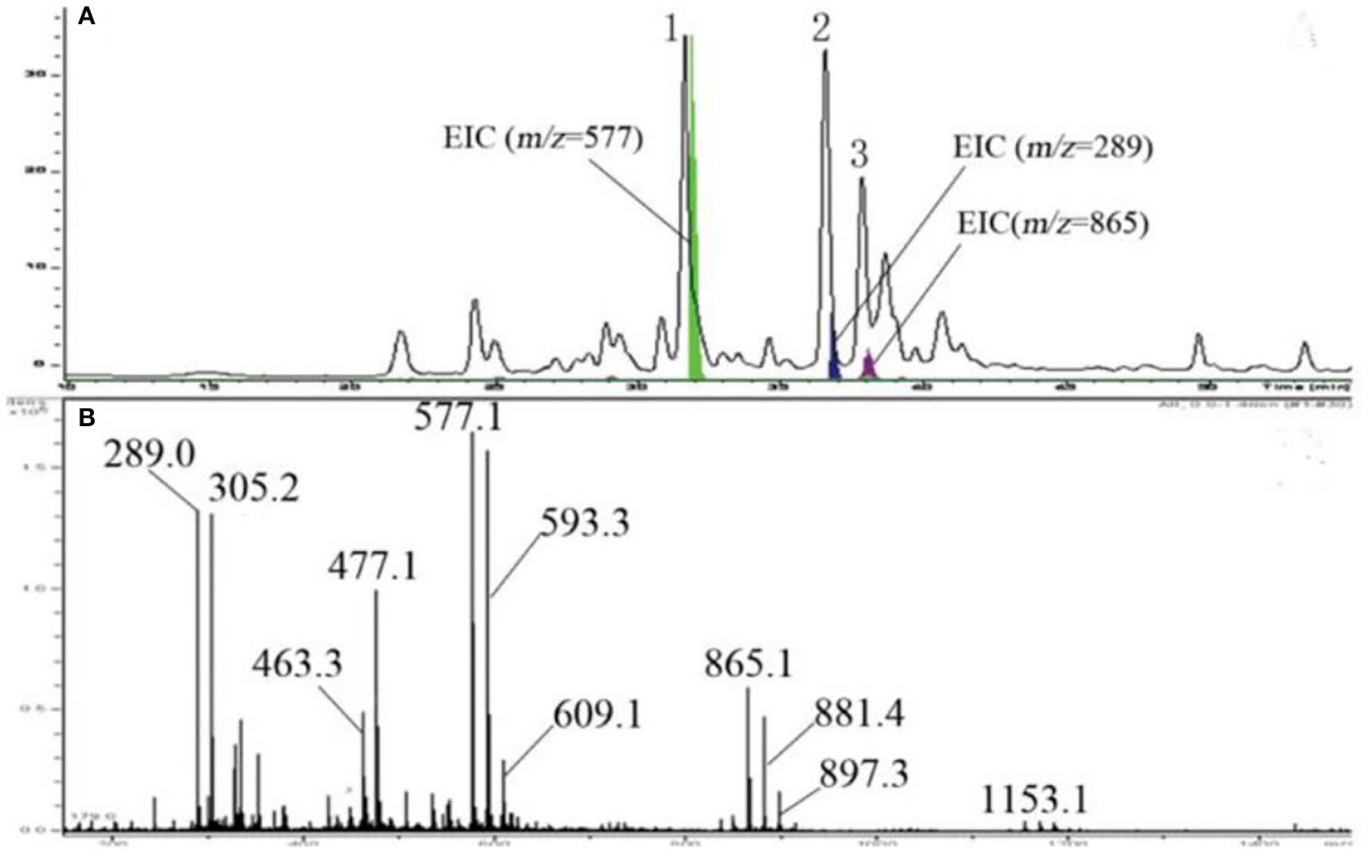
HPLC chromatogram, EICs **(A)**, and mass spectrum **(B)** of LSOPC using LC-ESI-MS.

Using the butanol-HCI assay, we found that LSOPC was rich in procyanidin. The procyanidin content of LSOPC was 106.22 ± 0.46% compared to that in grape seeds. Grape seed procyanidin with a purity of more than 95% are used as a standard product. Our laboratory has uncovered the structural information of the procyanidin in lotus seedpods (28). The mean degree of polymerization of LSOPC was found to be 3.2. The terminal units in LSOPC were 74% catechin and 26% epicatechin. The extension units in LSOPC were 26% catechin, 43% epicatechin, and 31% epigallocatechin. According to the HPLC chromatogram of LSOPC (Figure 1A), peaks 1, 2, and 3 exhibited maximum absorption at 279 nm and corresponded to extracted ion current chromatogram peaks of m/z 577, 289, and 865, respectively, suggesting that peaks 1, 2, and 3 represented procyanidin dimers, monomers (i.e., catechin or epicatechin), and trimers, respectively.

By coeluting procyanidin B1, B2, and B3, peak 1 was confirmed to be procyanidin B3. From the ESI-MS spectrum of LSOPC (Figure 1B), the compounds in LSOPC could be tentatively identified by their m/z ratios as catechin or epicatechin (m/z 289), gallocatechin or epigallocatechin (m/z 305), quercetin glycoside (m/z 463), quercetin glucuronide (m/z 477), procyanidin dimers (m/z 577.1), proanthocyanidin dimer gallate (m/z 593.3), prodelphinidin dimers (m/z 609.1), procyanidin trimers (m/z 865.1), and proanthocyanidin trimers with 1 gallocatechin or epigallocatechin unit and 2 catechin units (m/z 881.4), proanthocyanidin trimers with 2 gallocatechin or epigallocatechin units and 1 catechin or epicatechin unit (m/z 897.3), and procyanidin tetramers (m/z 1153.1). Thus, LSOPC consists of proanthocyanidin monomers, dimers, trimers, and tetramers.

### The Effect of LSOPC on the Growth of *L. plantarum*

**Figure 2:**
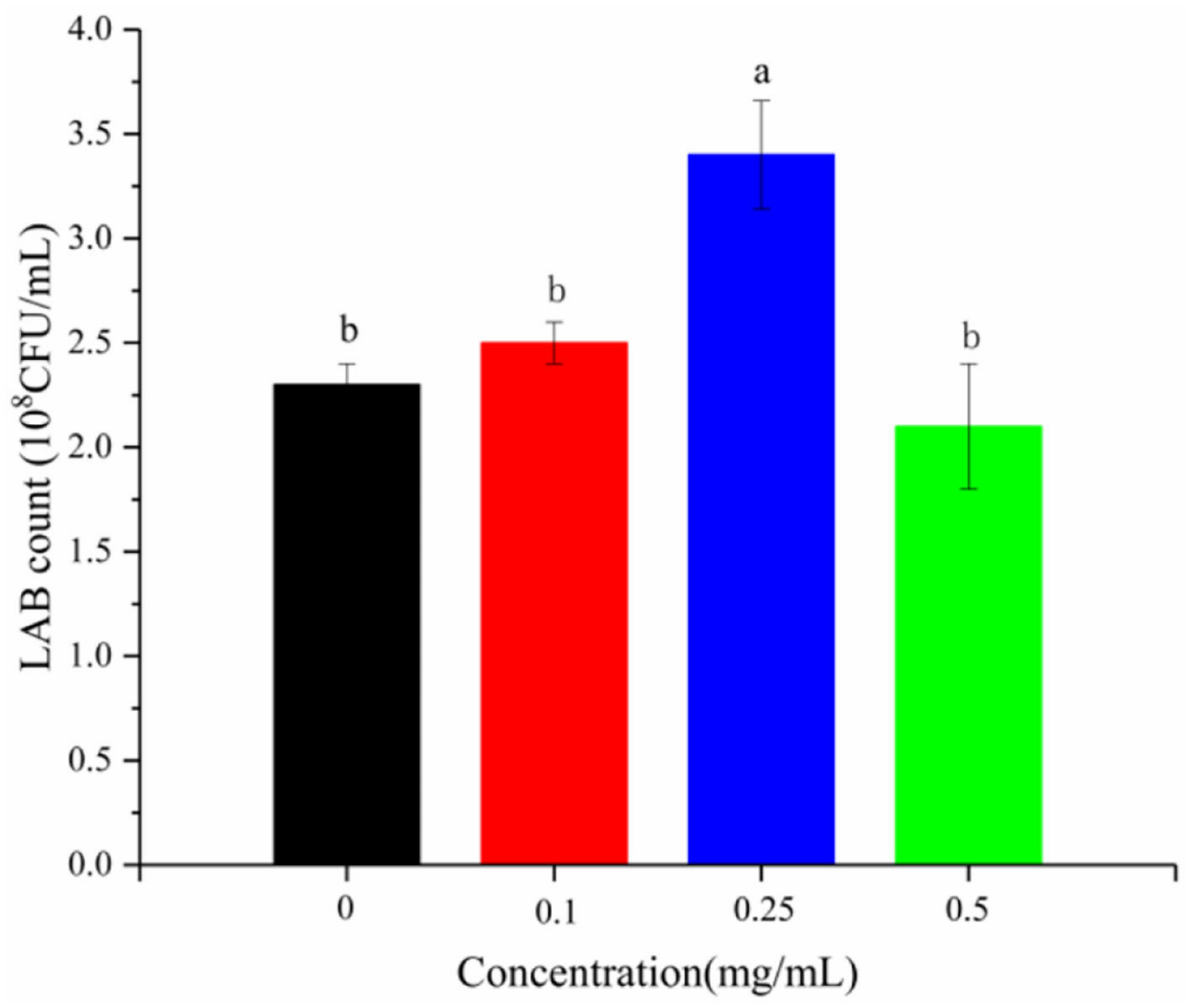
Content of LAB in yogurt with different concentrations of procyanidin. Different letters indicated significant differences (*P* < 0.05).

**Figure 3:**
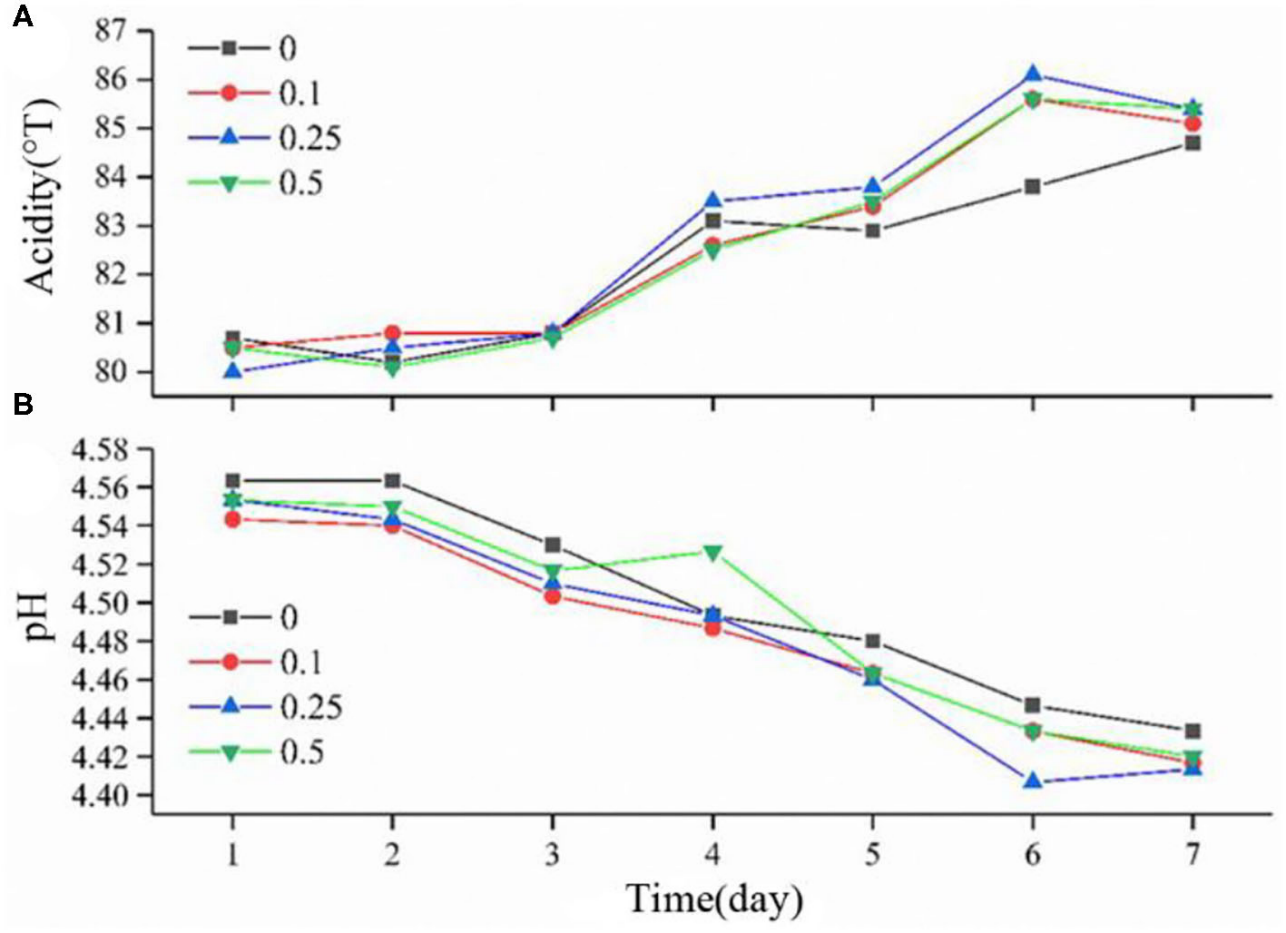
pH **(A)** and titratable acidity **(B)** of yogurt during 7 consecutive days.

Flavonoids inhibit the growth of some bacteria (29) and strictly control their subsequent metabolism (30). LSOPC was added to the medium of *L. plantarum* 21784 to verify this effect. LSOPC was not found to have a growth-inhibiting effect (Figure 2). On the contrary, it had a growth-promoting effect at 0.25 mg/mL. Moreover, pH and titratable acidity both correspond to the growth rate of bacteria (31). There was little increase in the pH and titratable acidity after adding LSOPC (Figure 3).

### The Effect of LSOPC on Fluorescent AGEs and CML Formation

**Figure 4:**
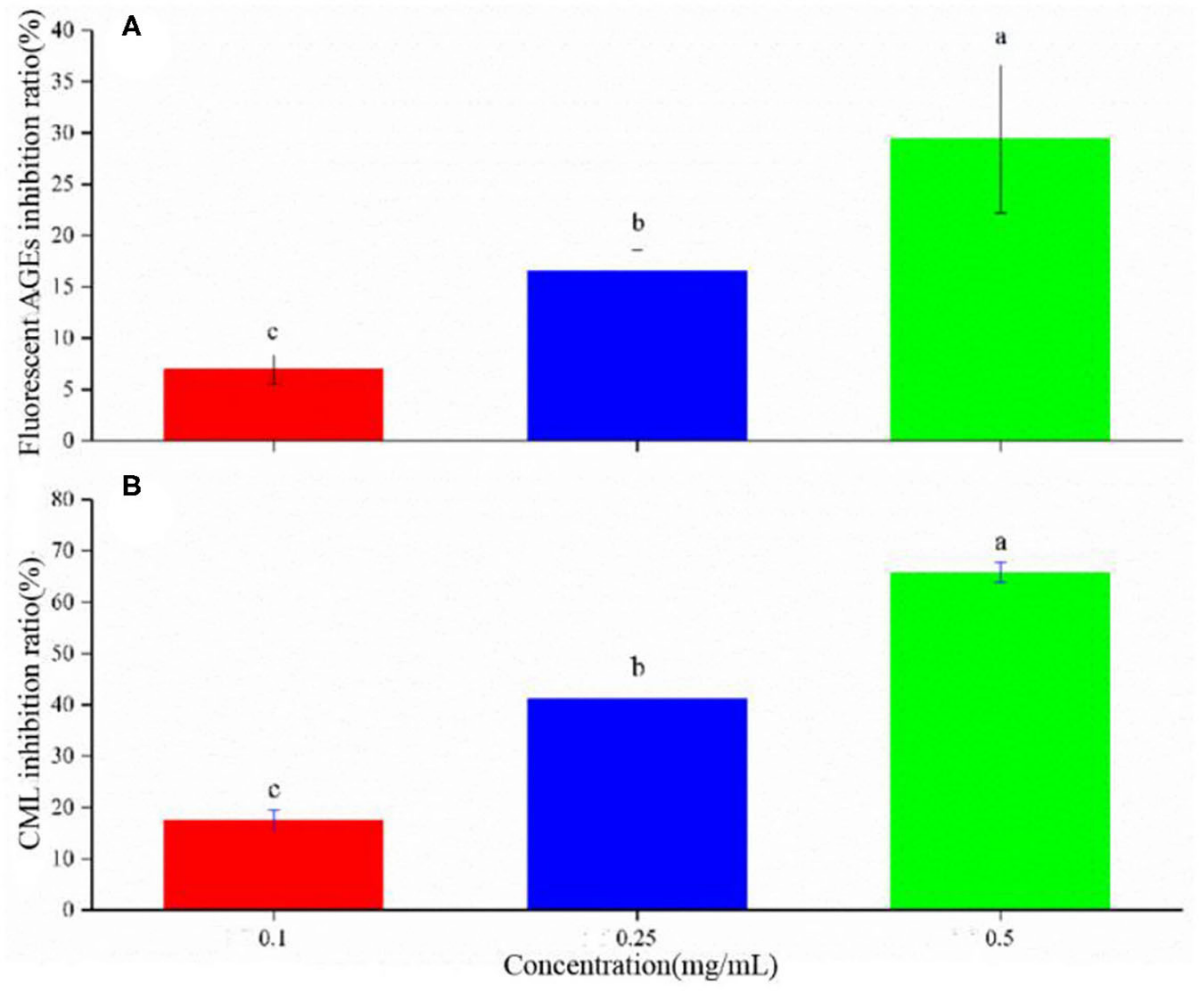
Inhibition rate of AGEs **(A)** and CML **(B)** in yogurt. Different letters indicated significant differences (*P* < 0.05).

Flavonoids can inhibit 2-dicarbonyl production and fluorescent AGE formation (32). The antioxidant activity is dependent on the number and location of hydroxyl groups on the aromatic ring (14, 33, 34). LSOPC could noticeably inhibit the formation of AGEs (Figure 4A). The increasing LSOPC concentrations from 0.1 to 0.5 mg/mL improved the inhibitory effect, increasing the inhibition rate from 7.0 to 29.4%.

CML, formed through a reaction between glyoxal and lysine, is a typically harmful substance and an important index of AGEs (35). Procyanidin can inhibit the formation of CML in a model system and a real food system; however, the inhibitory effect varies with the pH values and heating temperatures (35). Here, LSOPC was observed to have a significant inhibitory effect on the formation of CML in yogurt (Figure 4B). With increasing LSOPC concentrations of 0.1–0.5 mg/mL, the inhibition rate was increased from 17.5 to 65.8%. These data were consistent with the fluorescent AGE content detected in yogurt with different LSOPC concentrations.

### The Effect of Yogurt System on Procyanidin Degradation

**Figure 5:**
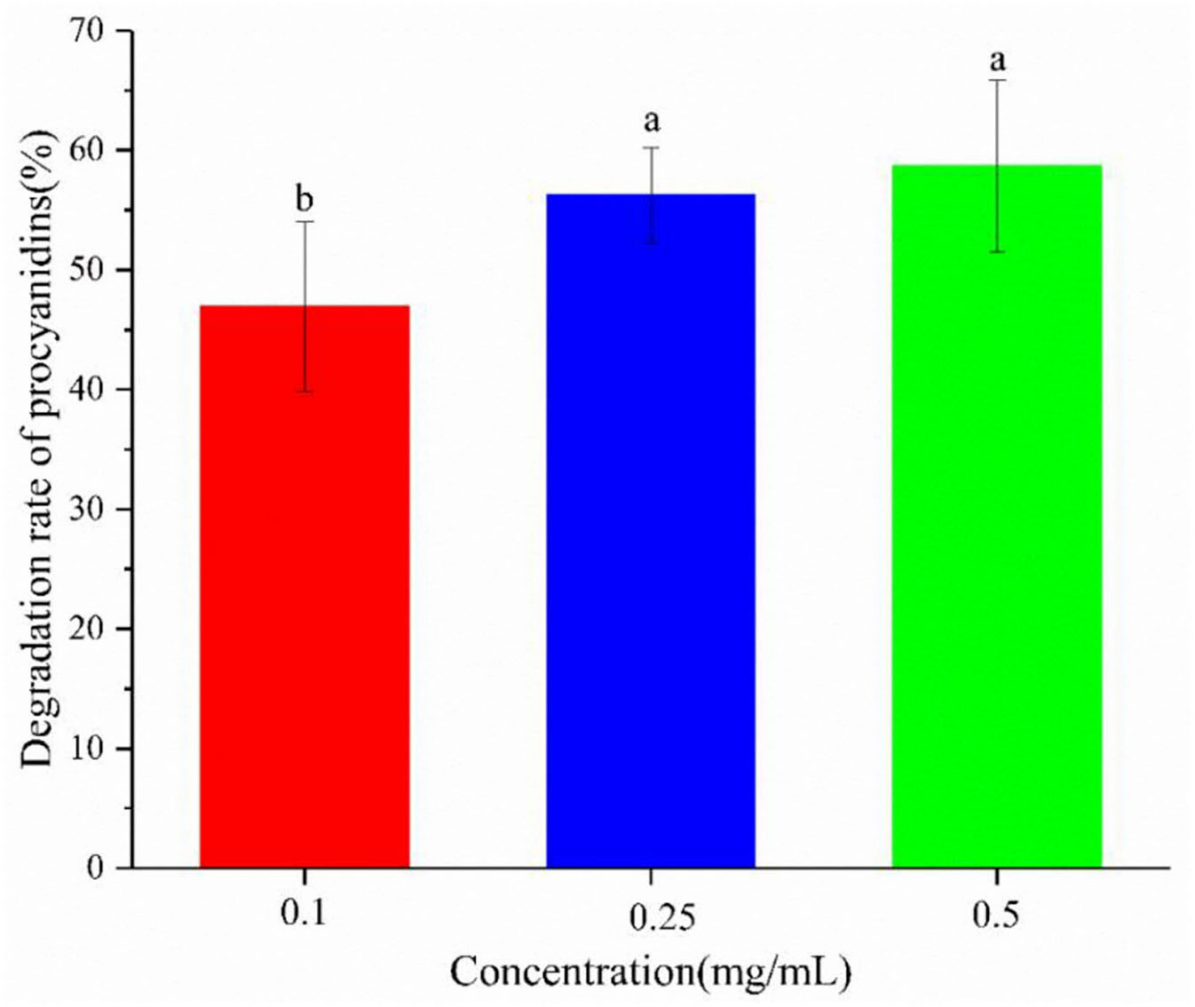
Degradation rate of procyanidin in the yogurt-procyanidin groups. Different letters indicated significant differences (*P* < 0.05).

The inhibitory effect of procyanidin on AGE formation is weakened by yogurt (4, 7, 35). The underlying mechanism was examined by studying the degradation of procyanidin in yogurt (Figure 5). On one hand, the procyanidin degradation rate was high and up to 50%, partly because procyanidin were unstable and degraded by LAB. On the other hand, procyanidin degradation could increase as its concentration increased, especially at lower concentrations of 0.1–0.25 mg/mL. At higher concentrations, procyanidin were more unstable, indicating a greater inhibition of procyanidin. To further explore the weakening of the inhibitory effect of procyanidin by yogurt, the degradation products of procyanidin were analyzing.

### Effect of Procyanidin on the Antioxidant Activity of Yogurt

**Figure 6:**
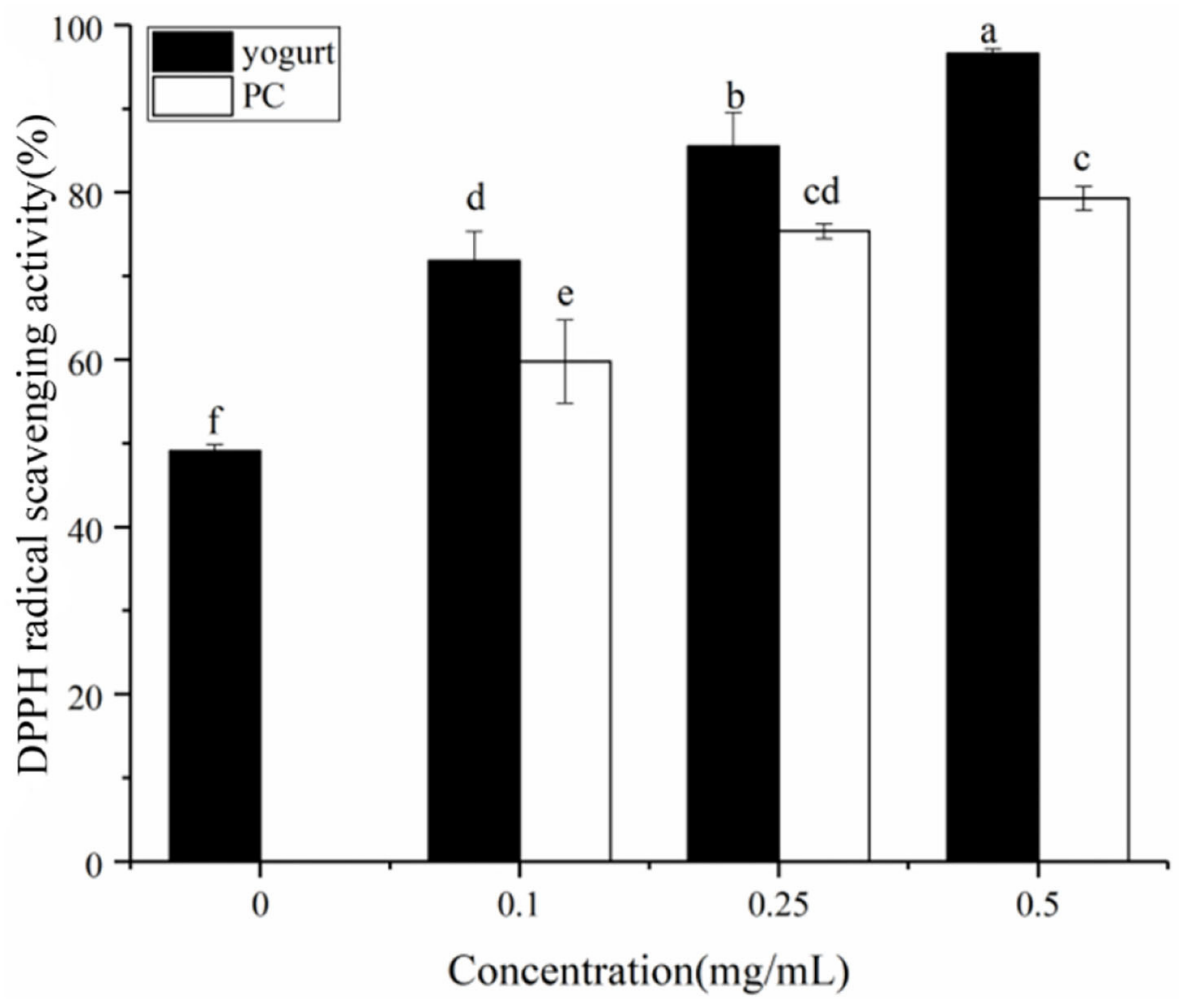
DPPH free radical scavenging by yogurt containing procyanidin and yogurt without procyanidin (solid cylinder). The free radical scavenging rate of procyanidin themselves was also presented as a control (hollow cylinder). Different letters indicated significant differences (*P* < 0.05).

Phenolic compounds have antiglycative activities due to their dicarbonyl-trapping capacity, free radical scavenging activity, and metal-chelating and antioxidant properties (36, 37). The DPPH free radical scavenging rate of yogurt was studied (solid cylinder, Figure 6); the scavenging rate was significantly higher after adding procyanidin. Also, at higher concentrations of added procyanidin, the antioxidant activity the stronger. The antioxidant capacity of flavonoids is closely related to the inhibition rate of the Maillard reaction (38). The effect of procyanidin on the antioxidant activity of yogurt also appeared correlated with its rate of AGE inhibition. Also, interestingly, a synergistic effect of antioxidant activity was found in the yogurt-procyanidin groups, whose scavenging rates were higher than in the procyanidin-only groups (hollow cylinder, Figure 6).

### Low-Field NMR Properties

**Figure 7:**
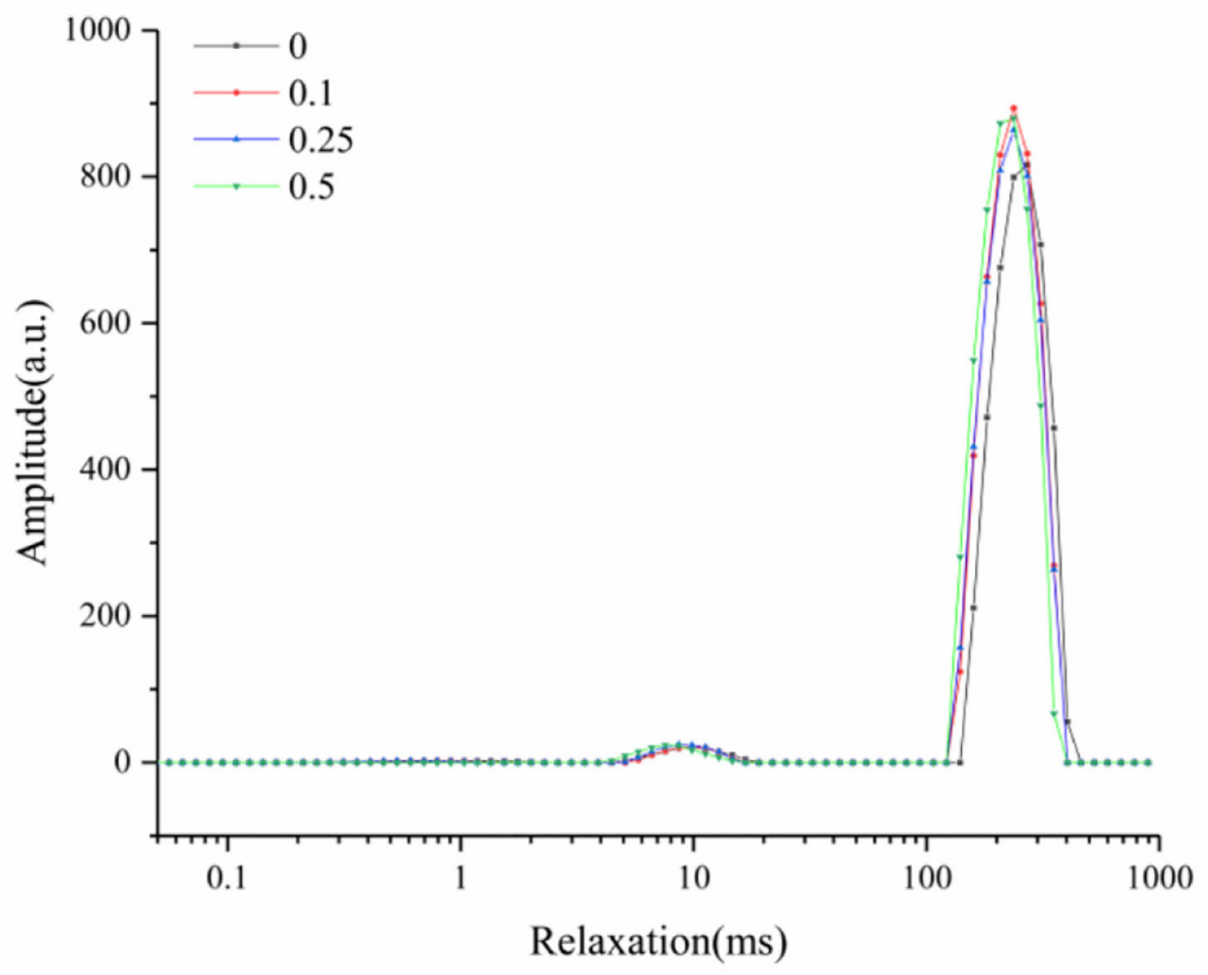
Low-field NMR spectra of yogurt.

The intermediates of fermented milk and cheese are similar; they are both non-membrane colloidal structures formed by the coagulation of proteins and polysaccharides. Three peaks in the T_2_ relaxation curves were ascribed to bound water (1–10 ms), immobile water (10–100 ms), and free water (100–1,000 ms), respectively. The relaxation peak of yogurt slightly shifted to the left with the increasing concentrations of added procyanidin (Figure 7), indicating that the free water in yogurt was more likely to bound with procyanidin. According to the polyhydroxy structure, procyanidin will provide more hydrogen bonding sites to control the free water in yogurt (3).

### Rheological Properties

**Figure 8:**
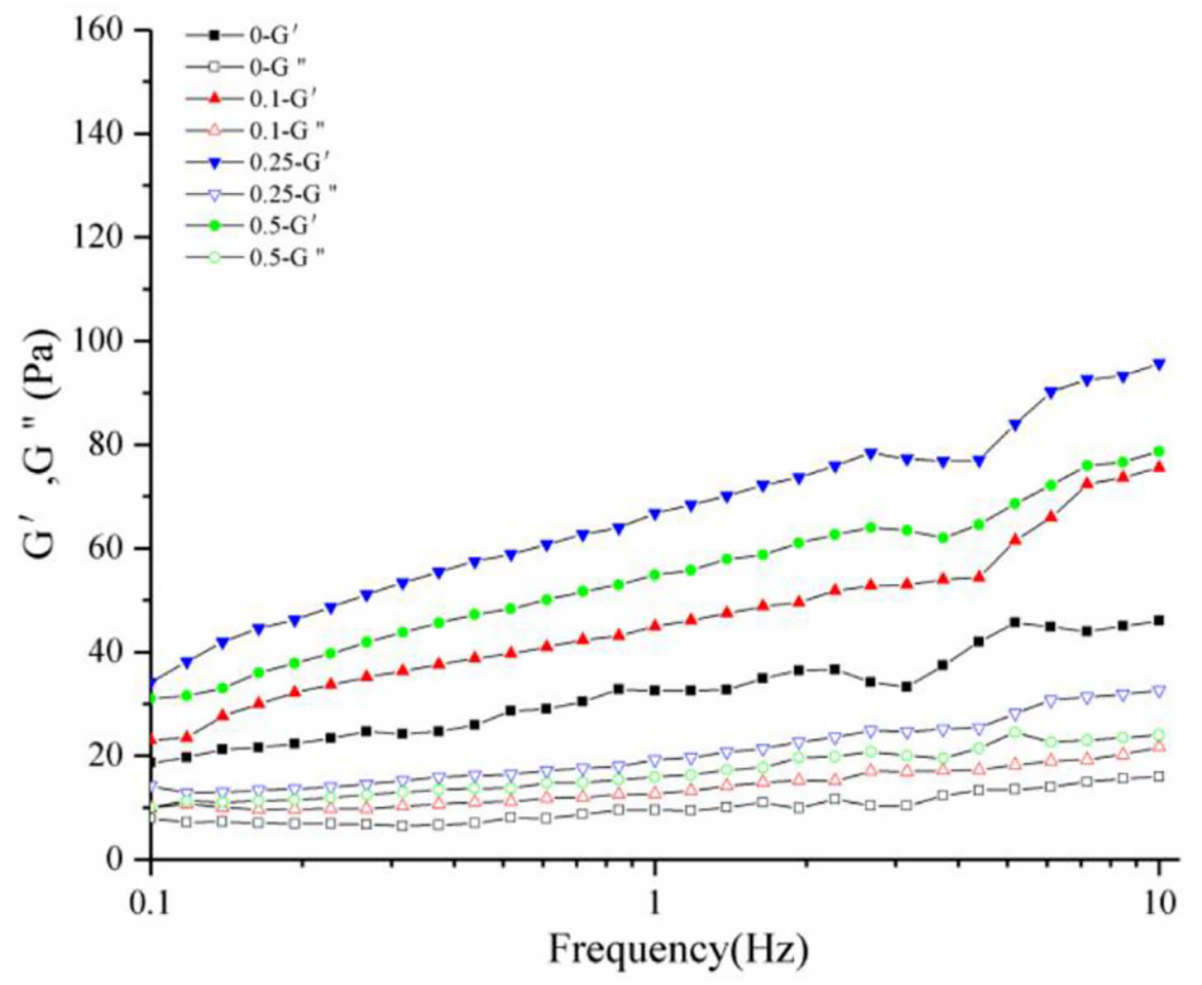
Dynamic rheological diagram of yogurt. G' is the elastic modulus, G" is the viscous modulus.

The rheological properties of a substance can be analyzed by comparing the storage (G') to the loss modulus (G”) master curves (39). The variation of G' and G” with frequency provides valuable information on the change in the stiffness and damping capability of a substance, respectively. G' and G” were measured for the frequency sweep tests (Figure 8). The G' values showed a substantial increase and a comparatively higher frequency dependence. The changes in G” curves were negligible compared to that in the G' curves, indicating that the yogurt acquired solid-like properties after the addition of procyanidin and had the characteristics of fermented milk.

**Figure 9:**
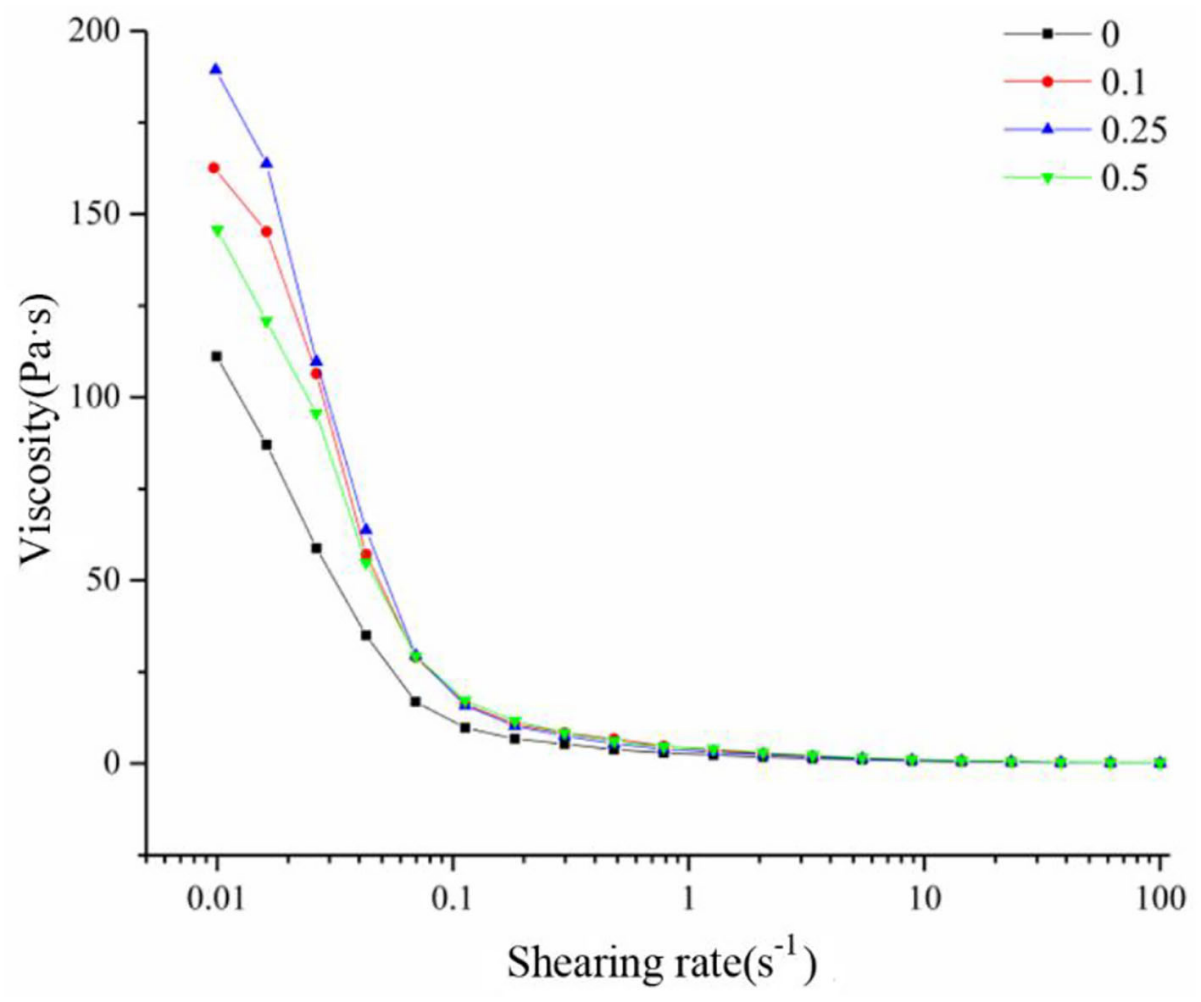
Static rheological diagram of yogurt.

Shear viscosity was also measured using a rheometer at a shear rate of up to 100 s^−1^. The viscosity significantly decreased at a very low shear rate, and then less affected at higher shear rate (Figure 9), consistent with the rheological properties of pseudoplastic fluids.

### Texture Properties

The sensory quality of yogurt is very subtle and can be influenced by many factors. General evaluation tools are needed in the industry to meet the global consumer demands for specific sensory attributes. The objective measurement of texture properties may well be far less complex than that of other sensory quality. The texture profile, including parameters like hardness, adhesiveness, springiness, cohesiveness, gumminess, chewiness, and resilience, of the yogurt samples, was analyzed (Table 1). The addition of procyanidin significantly increased the hardness, adhesiveness, gumminess, and chewiness. However, it did not affect the springiness, cohesiveness, or resilience of the yogurt samples, likely due to the increase of bound water content in yogurt. Similar results were observed when grapeseed oil was added to yogurt (40). The improvement of these parameters within a reasonable range should enhance the sensory quality of yogurt.

**Table 1:** Texture properties of yogurt with different concentrations (0, 0.1, 0.25, and 0.5 mg/ml) of procyanidin.

**Samples**	**Hardness**	**Adhesiveness**	**Springiness**	**Cohesiveness**	**Gumminess**	**Chewiness**	**Resilience**
0	7.89 ± 0.9^c^	28,357 ± 833^c^	0.97 ± 0.004^a^	0.729 ± 0.024^a^	5.53 ± 0.61^c^	5.37 ± 0.61^c^	0.052 ± 0.009^a^
0.1	12.4 ± 1.5^b^	34,263 ± 318^b^	0.96 ± 0.002^a^	0.703 ± 0.007^a^	8.08 ± 0.92^b^	7.82 ± 0.90^b^	0.042 ± 0.003^a^
0.25	15.7 ± 0.6^a^	58,267 ± 769^a^	0.97 ± 0.003^a^	0.713 ± 0.016^a^	10.3 ± 0.55^a^	10.1 ± 0.55^a^	0.040 ± 0.003^a^
0.5	13.0 ± 1.7^b^	34,027 ± 707^b^	0.96 ± 0.002^a^	0.712 ± 0.012^a^	8.66 ± 1.08^b^	8.39 ± 1.06^b^	0.041 ± 0.002^a^

*The data are given as mean ± S.D (n = 3). Different letters indicated a significant difference (p < 0.05)*.

### Volatile Flavor

Nineteen volatile compounds were identified by GC-MS from the yogurt samples, accounting for 37–46% of the absolute dry weight of the samples (Table 2). These compounds included two ketones, seven acids, five alkanes, and five olefins. The ketone content in the yogurt-procyanidin groups was noticeably higher than that in the control group. The content of 2-heptanone increased from 5.09 to 9.24% by adding 0.25 mg/mL procyanidin. The content of 2-nonanone increased from 2.40 to 3.12% as well. Ketone compounds can promote the degradation of AGE intermediate products (4). Thus, procyanidin likely control the formation of AGEs by increasing the content of ketone compounds.

**Table 2:** Flavor components in yogurt identified by GC-MS analysis.

**No**.	**RT**	**Component**	**Relative content (%)**			
			**0**	**0.1**	**0.25**	**0.5**
1	6.242	2-Heptanone	5.09	7.95	9.24	7.08
2	9.603	2-Nonanone	2.4	2.66	3.12	3.07
3	9.144	Heptanoic acid	0.54	0.69	0.74	0.82
4	10.898	Octanoic acid	0.99	1.21	1.76	1.33
5	16.256	n-Hexadecanoic acid	13.88	16.07	18.25	15.54
6	15.687	Tetradecanoic acid	2.05	2.21	1.98	1.72
7	16.404	Hexadecanoic acid	2.17	2.16	1.91	1.73
8	17.996	cis-10-Heptadecenoic acid	ND	1.43	1.47	1.82
9	19.021	(E)-9-Octadecenoic acid	1.33	2.24	2.21	1.98
10	12.093	Pentadecane	0.67	0.76	0.42	0.48
11	14.301	Cyclohexadecane	1.08	ND	ND	ND
12	16.343	Hexadecane	1.21	1.54	0.98	ND
13	15.649	Octadecane	2.16	1.85	1.39	ND
14	11.977	Eicosane	1.6	ND	ND	ND
15	8.691	D-Limonene	ND	0.5	0.45	0.38
16	18.266	1-Octadecene	0.32	0.89	0.75	0.44
17	19.113	1-Heptadecene	ND	0.69	0.88	0.36
18	13.101	Nonacos-1-ene	0.43	0.25	0.36	0.18
19	15.336	13-Methyl-Z-14-nonacosene	1.26	ND	ND	ND

**Figure 10:**
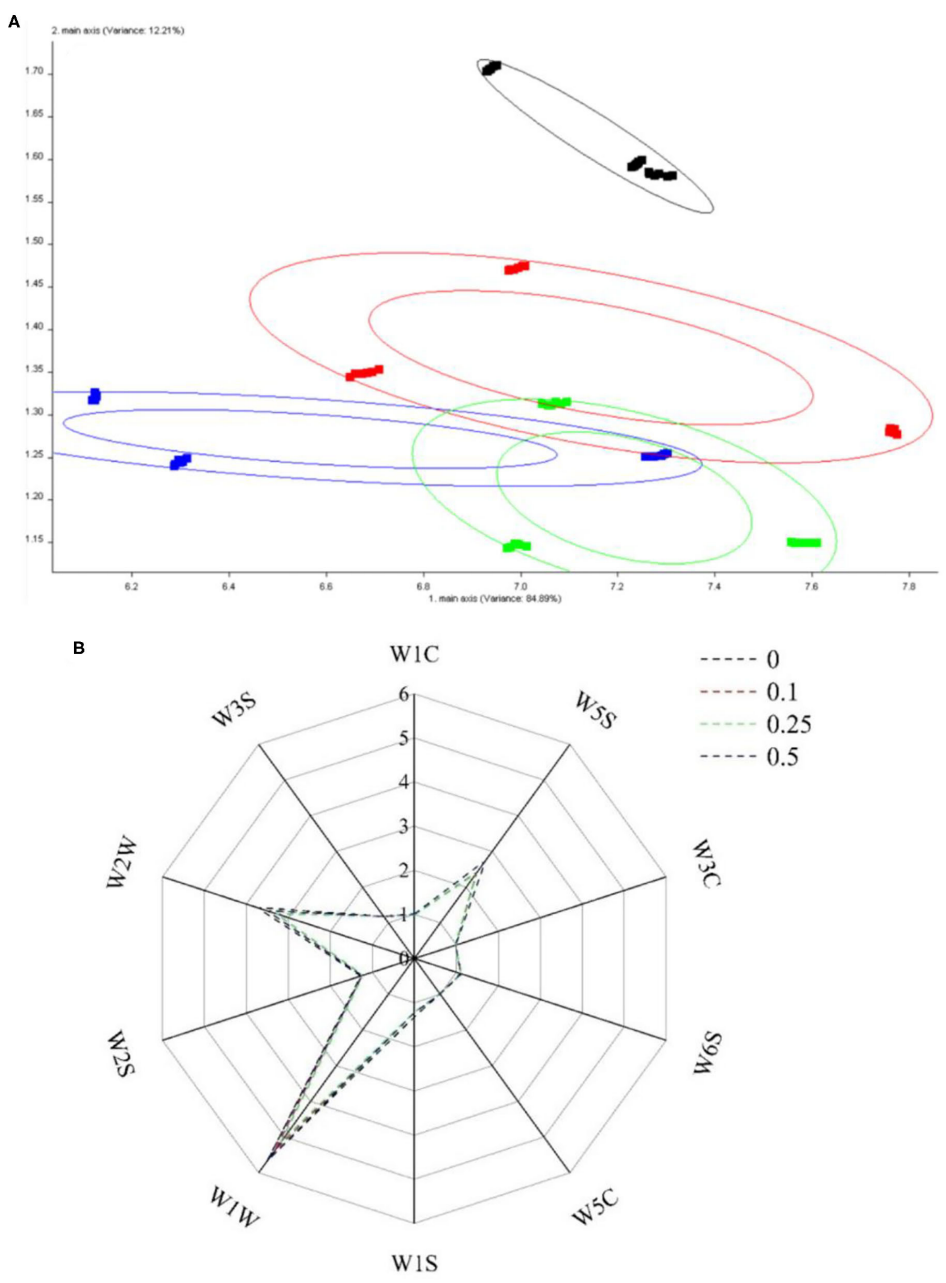
PCA diagram **(A)** and radar chart **(B)** of electronic nose.

The principal component analysis was performed by WinMuster software of the e-nose. The contribution rates of the first and the second principal components were 84.89 and 12.21%, respectively, and the total contribution rate was 97.10% (Figure 10A). These data indicated that the principal component could reflect all the characteristics of the volatile odor of yogurt with or without procyanidin. It is generally believed that a data point farther away from the origin has a greater contribution rate (41). Besides, the three yogurt samples with procyanidin displayed some differences and could be completely separated from the yogurt sample without procyanidin.

According to the radar chart (Figure 10B), the four yogurt samples displayed significantly different odor characteristics. All the samples had higher response values at sensors W5S, W1W, and W2W; in addition, the response values of the above sensors differed significantly. In comparison, the samples had similar response values at W6S. The odor characteristics from the analysis of the e-nose data were related to the composition of volatile compounds (GC-MS results).

### Organic Acids

**Figure 11:**
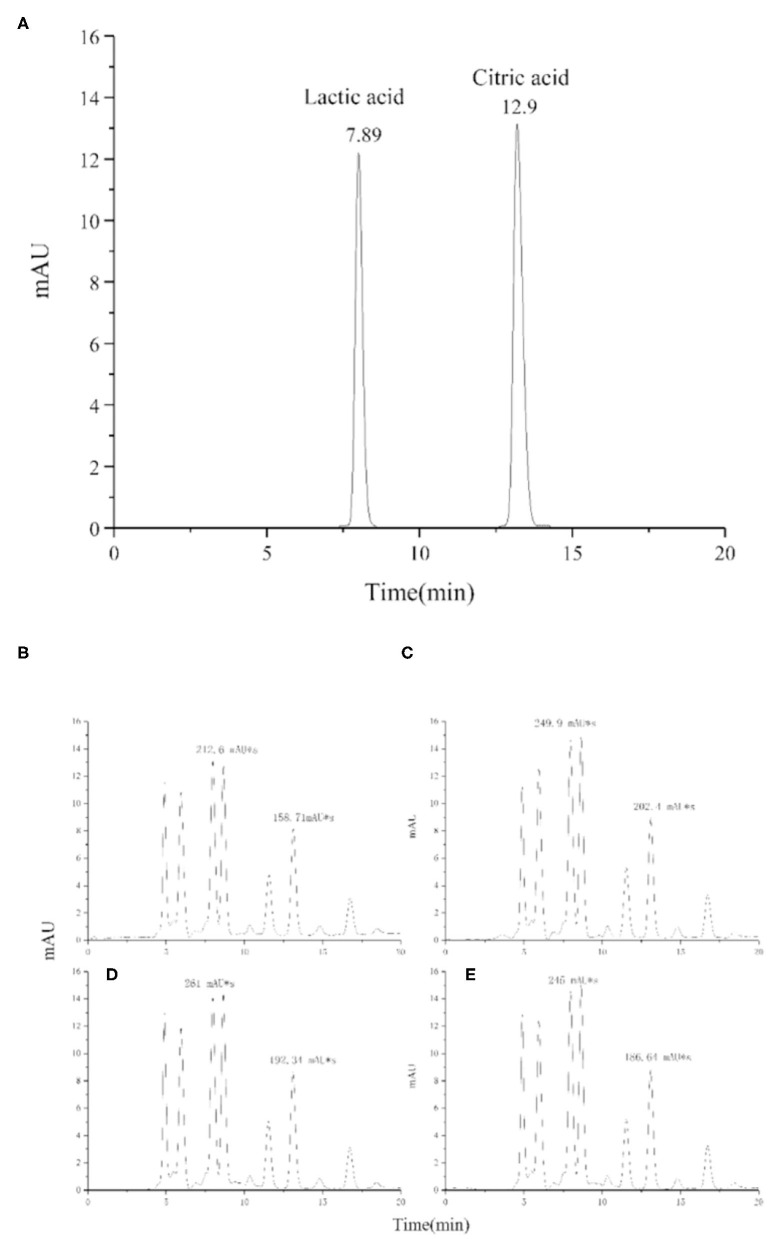
HPLC chromatogram of lactic acid and citric acid in standard sample **(A)** and yogurt samples, 0 mg/mL procyanidin **(B)**, 0.1 mg/mL procyanidin **(C)**, 0.25 mg/mL procyanidin **(D)**, and 0.5 mg/mL procyanidin **(E)**.

Organic acids in fermented dairy products play an important role as natural preservatives and contribute to the characteristic sensory properties of the product (42). Lactic acid content affects, more than other organic acids present in lower amounts, the “sourness” intensity of yogurt, which is disliked by some customers. The mean concentration of lactic acid was increased from 1.3 to 1.6 mg/mL by adding procyanidin (Figure 11 and Table 3); however, the differences were very small in the yogurt-procyanidin groups.

**Table 3:** Content of lactic acid and citric acid in yogurt different concentrations (0, 0.1, 0.25, and 0.5 mg/ml) of procyanidin.

**Samples**	**Lactic acid (mg/mL)**	**Citric acid (mg/mL)**
0	1.34 ± 0.1^b^	0.29 ± 0.022^b^
0.1	1.6 ± 0.11^a^	0.36 ± 0.009^a^
0.25	1.67 ± 0.07^a^	0.34 ± 0.009^a^
0.5	1.56 ± 0.06^a^	0.33 ± 0.016^a^

*The data are given as mean ± S.D (n = 3). Different letters indicated a significant difference (p < 0.05)*.

Citric acid is usually present in milk as a product of bovine metabolism. Citric acid is also known to be utilized during the fermentation process, but it is underutilized in the storage process (43). Here, the yogurt-procyanidin groups had a higher concentration of citric acid than the control group. The citric acid concentration was increased from 0.29 to 0.36 mg/mL by the addition of procyanidin. The increase in organic acid concentration in yogurt-procyanidin groups was in agreement with the decrease in pH value.

## Conclusion

Due to the increasing global demand for food function, studying natural additives is necessary. Interestingly, LSOPC demonstrate an inhibitory effect on AGE formation in a simulated system. The increasing LSOPC concentrations from 0.1 to 0.5 mg/mL improved the inhibitory effect, increasing the inhibition rate from 7.0 to 29.4%. At higher concentrations of added procyanidin, the antioxidant activity the stronger. In this study, yogurt with high lactose and protein content was used to evaluate the inhibitory effect of LSOPC. Firstly, LSOPC were found to improve the antioxidant properties of yogurt and reduce the consumption of ketones, thereby displaying an inhibitory effect against AGE formation. Besides, the increase in the bound water content, viscosity, and flavor of yogurt was found in the yogurt-procyanidin groups. These findings likely advance the value-added applications of procyanidin in food additives.

## Data Availability Statement

The original contributions presented in the study are included in the article/supplementary material, further inquiries can be directed to the corresponding author/s.

## Author Contributions

NF: conceptualization, methodology, investigation, and writing-original draft. YS: data curation and writing-original draft. CH: methodology. JT: data curation. ZH and CW: supervision. ZG: conceptualization. QW: writing-review and editing, supervision, and funding acquisition. JX: conceptualization and funding acquisition. All authors contributed to the article and approved the submitted version.

## Funding

This work was financially supported by National Natural Science Foundation of China (Grant Nos. 32001705 and 21908048), Key Laboratory of Food Nutrition and Functional Food of Hainan Province (No. KF202009), State Key Laboratory of Marine Resource Utilization in South China Sea (Hainan University) (No. MRUKF2021002), and the Collaborative Grant-in-Aid of the HBUT National 111 Center for Cellular Regulation and Molecular Pharmaceutics (Nos. XBTK-2021003 and XBTK-2020005).

## Conflict of Interest

The authors declare that the research was conducted in the absence of any commercial or financial relationships that could be construed as a potential conflict of interest.

## Publisher's Note

All claims expressed in this article are solely those of the authors and do not necessarily represent those of their affiliated organizations, or those of the publisher, the editors and the reviewers. Any product that may be evaluated in this article, or claim that may be made by its manufacturer, is not guaranteed or endorsed by the publisher.
